# Growth and Coalescence of 3C-SiC on Si(111) Micro-Pillars by a Phase-Field Approach

**DOI:** 10.3390/ma12193223

**Published:** 2019-10-01

**Authors:** Marco Masullo, Roberto Bergamaschini, Marco Albani, Thomas Kreiliger, Marco Mauceri, Danilo Crippa, Francesco La Via, Francesco Montalenti, Hans von Känel, Leo Miglio

**Affiliations:** 1 L-NESS and Department of Materials Science, Università di Milano-Bicocca, via R. Cozzi 55, I-20125 Milano, Italy; 2 Laboratory for Solid State Physics, ETH Zürich, Otto-Stern-Weg 1, CH-8093 Zürich, Switzerland; 3 LPE S.P.A., Sedicesima Strada, I-95121 Catania, Italy; 4 LPE S.P.A., Via Falzarego, I-20021 Baranzate, Milano, Italy; 5 CNR-IMM, Strada VIII 5, I-95121 Catania, Italy

**Keywords:** 3C-SiC, micro-crystals, epitaxy, morphology, phase-field, kinetic growth

## Abstract

3C-SiC is a promising material for low-voltage power electronic devices but its growth is still challenging. Heteroepitaxy of 3C-SiC on Si micrometer-sized pillars is regarded as a viable method to achieve high crystalline quality, minimizing the effects of lattice and thermal expansion mismatch. Three-dimensional micro-crystals with sharply-faceted profiles are obtained, eventually touching with each other to form a continuous layer, suspended on the underlying pillars. By comparing experimental data and simulation results obtained by a phase-field growth model, here we demonstrate that the evolution of the crystal morphology occurs in a kinetic regime, dominated by the different incorporation times on the crystal facets. These microscopic parameters, effective to characterize the out-of-equilibrium growth process, are estimated by a best-fitting procedure, matching simulation profiles to the experimental one at different deposition stages. Then, simulations are exploited to inspect the role of a different pillar geometry and template effects are recognized. Finally, coalescence of closely spaced crystals ordered into an hexagonal array is investigated. Two possible alignments of the pattern are compared and the most convenient arrangement is evaluated.

## 1. Introduction

Silicon carbide (SiC) is a highly regarded semiconductor material for applications in power electronics [1], due to the wide band gap (2.3 to 3.2 eV). While the hexagonal 4H- and 6H-SiC allotropes are exploited commercially, the cubic 3C-SiC is still object of intense research as it offers some key advantages [2]. In particular, its 2.3 eV band gap would enable applications in the low voltage range of 600–1200 V, delivering low resistance and good electron mobilities [3]. However, growing high-quality 3C-SiC is an open issue [2].

A convenient path is to exploit its crystallographic compatibility with silicon (Si) by heteroepitaxy. This way, a reduction in the cost of the processing is possible, profiting of the well-established Si technology. Direct deposition of 3C-SiC on a bare Si wafer is practically doable only for sub-micron-thin layers. The large misfit in lattice parameter and thermal expansion coefficients, respectively ∼20% and ∼8%, hinders any practical application of thicker deposits as resulting in a highly defective material with large wafer bowing and cracking [4]. Several options are being explored nowadays to minimize these issues [5,6]. Among them, a promising approach consists of growing the SiC film suspended on top of micrometer sized Si pillars, deeply etched into the substrate. It has been shown, indeed, that such an architecture is very effective in relieving strain by exploiting the additional degree of freedom of pillar tilting and rotation [7], allowing for a significant reduction of wafer bowing even with several μm thick SiC layers [8].

The growth process can be divided in two stages. In the first, individual crystals develop on top of the Si pillars. The three-dimensional (3D) growth typically results into high-aspect ratio faceted morphologies that can conveniently relax strain by plasticity. Defects can possibly be expelled through the crystal sidewalls so to achieve high crystal quality in the upper part [9,10]. The method has been successfully applied to several heteroepitaxial systems such as Ge/Si [11,12,13], GaAs/Si [14,15], GaAs/Ge/Si [16] and GaN/Si [17]. It has already been demonstrated also for 3C-SiC/Si in References [9,18], with beneficial effects on the crystal quality [18] and a substantial decrease in defectivity [19,20].

The second growth stage consists of the coalescence of the individual micro-crystals into a connected structure, eventually ending into a continuum film [21,22]. In order to achieve a smooth morphology, without holes and irregularities, surface diffusion has to be active [23] thus requiring the growth to be performed at high-temperature. Growth of 3C-SiC suspended layers, arranged into patches as large as hundreds of micrometers, was reported in References [8,19]. The reduced bowing made possible by the pillar architecture allowed for the growth of thick (up to 20 μm), single-crystal 3C-SiC layers with a significant reduction in stacking faults density compared to the case of unpatterned substrates [9].

Understanding the crystal evolution is of primary importance to optimize the growth process and select the most convenient pattern geometry. In this work we then performed an in-depth analysis of the growth and coalescence of 3C-SiC micro-crystals on Si(111) pillars by closely comparing dedicated experiments and computer simulations. The morphological evolution of the faceted shape of individual crystals is described in a kinetic growth regime [24,25], determined by facet-dependent incorporation times. These are obtained by a reverse-engineering strategy, by matching simulation results to the experimental profiles at different growth stages. Simulations are then exploited to inspect the role of the Si pillar geometry in templating the SiC crystal shape. Finally, different conditions of crystal coalescence are investigated, depending on the relative arrangement of the neighboring pillars.

## 2. Materials and Methods

### 2.1. Experimental

Si(111) substrates were patterned into hexagonal arrays of pillars on 4 in wafers. The pillars were obtained by standard Bosch process in the shape of hexagonal prisms 8 μm deep and 2 or 5 μm wide, separated with each other by 2 or 3 μm trenches. Pillars were partitioned into 100 μm hexagonal patches separated by 5 μm trenches to avoid their contact so to mitigate substrate bowing. 3C-SiC growth was performed in a horizontal hot-wall CVD reactor (ACISM10) with a large reaction chamber. The standard growth procedure consists of several steps. First, a bake out was done at 500 ${}^{\circ }$C under high vacuum (about 10${}^{4}$ mbar). Then, after a quick H${}_{2}$ etching of the Si surface, carbonization is initiated by introducing the ethylene (C${}_{2}$H${}_{4}$) gas in the reaction chamber while raising the temperature up to 1140 ${}^{\circ }$C. The carbonization temperature is maintained for 10 min and, when carbonization is completed, the temperature is raised to the growth temperature of 3C-SiC at 1370 ${}^{\circ }$C. A growth rate of 6 μm/h was set and the deposition of the 3C-SiC epitaxial layer was performed by letting C${}_{2}$H${}_{4}$ flow into the chamber together with trichlorosilane (TCS) in a low-pressure regime (100 mbar). Samples were grown for different durations so to obtain different deposition thicknesses. The morphological analysis was performed by Scanning Electron Microscopy SEM using a Zeiss Ultra 55 at electron beam voltage of 5 kV. Cross-sections were performed by a dual beam FIB/SEM Zeiss Nvision 40, using a Ga ion beam at 30 kV and up to 13 nA currents.

### 2.2. Phase-Field Model of Kinetic Growth

**Figure 1:**
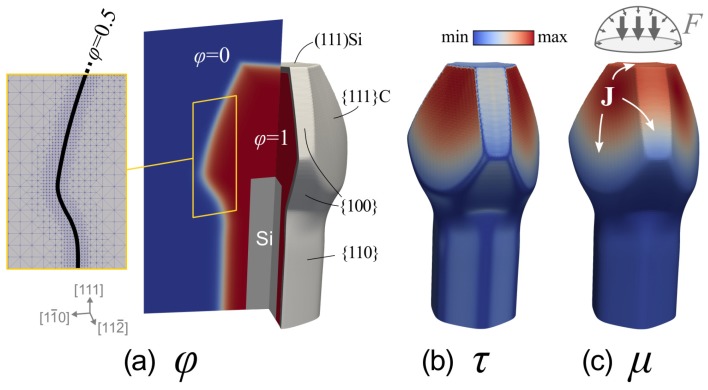
(**a**) Representation of the crystal structure in the phase-field model for a realistic, faceted morphology. The φ function is shown in a {$11\bar{2}$} cross section. The inset shows the adaptively refined mesh along with the contour line of the crystal surface (φ = 0.5). Colour map of (**b**) the adatom lifetime $\tau (\hat{n})$ and of (**c**) the chemical potential μ on the crystal surface, with the white arrows showing the diffusion direction of the material along the surface. A schematic representation of the flux distribution is also reported.

Simulations of the 3D growth of SiC micro-crystals on Si pillars are performed by means of the phase-field approach defined in Reference [26] and already successfully applied to model out-of-equilibrium epitaxy in Reference [27]. The key concept consists of the implicit definition of the system geometry by means of the phase-field function φ, valued 1 into the crystal and 0 in the surrounding vacuum, as illustrated in Figure 1a for a representative morphology. The free-surface is identified as the diffuse-interface region, of thickness ϵ, in between those two bulk phases and nominally localized by the φ = 0.5 isoline (see inset). The evolution of the surface shape is then traced by the temporal variation of the φ function itself (see Reference [28] for a review). Here, a combination of material deposition, with orientation dependent rate *F* and surface diffusion, driven by the gradient of adatoms chemical potential μ, is considered:

(1) $$\frac{\partial \varphi }{\partial t}=F\left(\hat{n}\right)\left|\nabla \varphi \right|+\nabla \cdot \left[M(\varphi )\nabla \mu \right],$$

with the surface normal vector $\hat{n}=-\nabla \varphi /\left|\nabla \varphi \right|$ and the mobility function $M\left(\varphi \right)=M_{0}\left(36/\epsilon \right)\varphi^{2}{\left(1-\varphi \right)}^{2}$ such to restrict motion within the (diffused) surface region only. The adatom chemical potential is defined according to Reference [29] as the summation of the variational derivative of free-energy *G* with respect to the φ variable and of a kinetic term proportional to the evolution rate ∂φ/∂t itself through the orientation dependent coefficient $\tau (\hat{n})$, playing the role of adatom incorporation time:

(2) $$g\left(\varphi \right)\cdot \mu =\frac{\delta G}{\delta \varphi }+\epsilon \tau \left(\hat{n}\right)\frac{\partial \varphi }{\partial t},$$

with $g\left(\varphi \right)=30\varphi^{2}{\left(1-\varphi \right)}^{2}$ a stabilizing function introduced for improving numerical convergence [30,31,32]. In the absence of deposition or in the case of close-to-equilibrium processes, μ is fully defined by the system energetics that, in the present case, just consists of surface energy. Full-strain relaxation (at the growth temperature) is assumed to happen by plasticity. Here we do the simplifying assumption of isotropic surface energy density γ which will be validated *a posteriori* by comparing simulations with experiments (see below). This way, the thermodynamic contribution is just proportional to the local profile curvature and takes the form $\delta G/\delta \varphi =\gamma \left[-\epsilon \nabla^{2}\varphi +\left(1/\epsilon \right)B^{\prime }\left(\varphi \right)\right]$, with double-well potential $B\left(\varphi \right)=18\varphi^{2}\left(1-\varphi^{2}\right)$ [28]. Incorporation times τs account for the effective lifetime of the adatoms on the surface before being permanently integrated in the growing structure. They represent the net result of many microscopic processes at the surface, involving the local dynamics of steps and defects and hence they are facet dependent. The faceting of the crystal shape is here enforced by such anisotropy of incorporation times τ. A convenient formula [33] is considered for defining the $\tau (\hat{n})$ function:

(3) $$\tau \left(\hat{n}\right)=\tau_{0}\sum_{hkl}\tau_{hkl}{\left(\hat{n}\cdot \left[hkl\right]\right)}^{\frac{1}{w}}\Theta \left(\hat{n}\cdot \left[hkl\right]\right)$$

The summation runs on all possible facets (hkl), each corresponding to a local maximum of incorporation time with amplitude $\tau_{hkl}$ (scaled by a factor $\tau_{0}$) and width ∼w. Here we consider {100}, {110} and {111} facet families as marked in Figure 1a. {111}-Si terminated facets and {111}-C terminated facets are distinguished thanks to the Heaviside function Θ which selects the specific orientation of the vector [hkl]. Figure 1b shows by color map the $\tau (\hat{n})$ function at the surface of the profile drawn in panel (a) while the resulting distribution of the chemical potential μ, responsible for material currents J, is reported in panel (c).

Simulation parameters were set to be consistent with the realistic system. The distribution of incoming material was modeled by considering a 70% vertical component, while the remaining 30% was made of an isotropic contribution coming from the hemispherical region above the sample (as sketched in Figure 1c). The nominal deposition rate of 6 μm/h was set for the {111}-Si terminated top facet. The isotropic surface energy density was γ = 20 eV/nm${}^{2}$ [34]. The length unit was set in μm to match the crystal sizes and a value of ϵ = 0.8 μm was used for the phase-field interface width. Simulation times were scaled to the unit of hours by suitably tuning mobility (here $M_{0}\approx$ 2.5·10${}^{6}$ nm${}^{6}$/(eV·s)). Adatom lifetimes were scaled accordingly by setting $\tau_{0}\approx$ 1 eV·s/nm${}^{4}$ (and width *w* = 0.01). The Si pillar geometry (see Figure 1a) was assumed as the initial profile. The AMDiS finite element method toolbox [35,36] was used for the numerical implementation, exploiting a semi-implicit integration time scheme and local mesh refinements to reach a maximum resolution at the crystal surface of the order of 80 nm. A cross-section view of the adaptive mesh is reported in the inset of Figure 1a.

## 3. Results and Discussion

**Figure 2:**
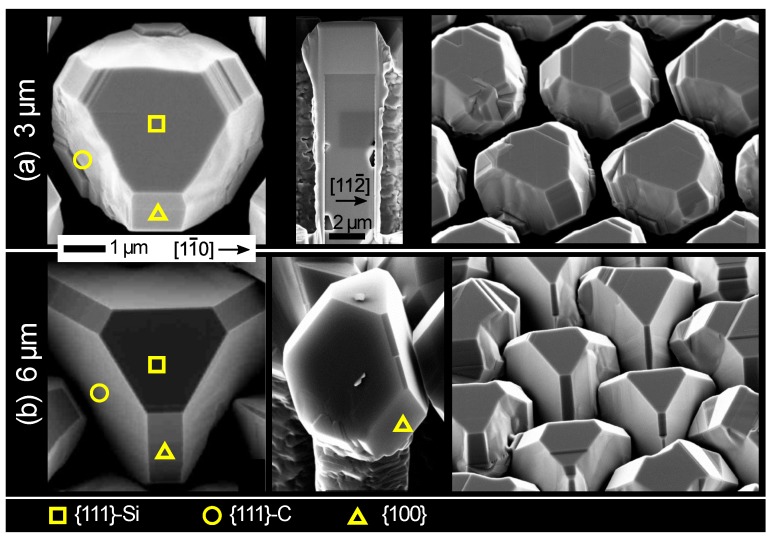
SEM views of SiC crystals grown on Si pillars after (**a**) 3 μm and (**b**) 6 μm of deposition. Si pillars consist of 8 μm tall hexagonal prisms with {110} sidewalls and 2 μm large {111} top facet and they are arranged in an hexagonal pattern with 3 μm spacing. The main crystal facets are marked by symbols.

The morphological evolution of 3C-SiC crystals has been inspected first experimentally by growing samples for different deposition thicknesses. Figure 2 shows Scanning Electron Microscope (SEM) views of the crystals obtained after deposition of (a) 3 and (b) 6 μm SiC for the same Si pillar pattern. A neat tendency to form sharp facets is recognizable since the beginning of the growth with a pronounced dominance of {111} facets (both Si- and C-terminated) and, smaller, {100} facets. First-principle surface energy calculations reported in Reference [34] indicate that such facets are indeed the most stable but the crystal morphology evolution here observed cannot be interpreted at all as an equilibrium shape. First, the (3 × 2) Si-terminated [37] reconstruction makes {100} facets the most stable at any C/Si growth condition so that those facets should have a greater extension in the equilibrium crystal shape [38], especially in Si-rich environment. This is the opposite of experiments, suggesting a progressive shrinkage of {100} planes at the advantage of the neighboring {111} facets as the growth proceeds from panel (a) to (b). Similarly, the area of the {111}-Si terminated crystal-top facet shrinks during growth, despite its surface energy should be lower with respect to the {111} C-terminated planes on the sides. Moreover, the surface energy of {110} facets is comparable to that of {111} ones, unless considering very Si-rich conditions, so that they should also take part in the crystal shape. Such discrepancies clearly suggest a significant distance of the experimental growth conditions from equilibrium, altering the relative facet extensions beyond the prediction of a simple energetic criterion.

Kinetic effects due to the out-of-equilibrium nature of the deposition process are then to be considered in order to explain the observed morphologies [27]. To this purpose, it is key to compare the advancing of each facet on the growth front in the two stages of Figure 2. Evidently, {111} C-terminated facets are those growing larger in size, depressing the expansion of the {100} planes at the crystal sides and consuming the {111} Si-terminated top facet. In the framework of the kinetic Wulff construction [24,39], the facets appearing in the crystal shape are the ones with lower growth rate: the lowest is the facet velocity, the more it develops in size. All intermediate orientations grow faster and remain excluded from the profile. Then, {111} and {100} facets correspond to local minima in growth rate function, with {111} C-terminated facets being the slowest, as remarked by the limited lateral expansion compared to the vertical growth in the experimental profiles.

As widely discussed in Reference [26], a general kinetic model for the growth of the faceted crystal can be based on the definition of adatom incorporation lifetimes τ, rather than on a mere fitting of experimental growth velocities. This is quite convenient as τs are intrinsic, yet effective, properties of the facet themselves, independent of the actual geometry and substrate patterning but able to return facet growth rates which dynamically depend on the actual redistribution of the adatoms between the neighboring facets.

**Figure 3:**
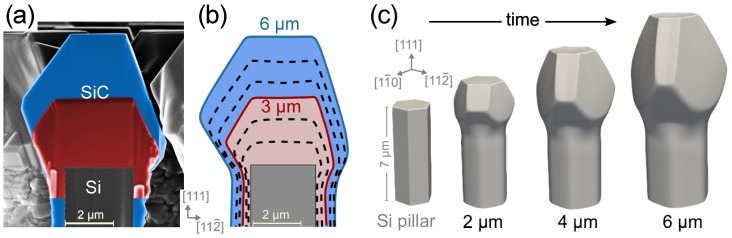
(**a**) Superposition of SEM cross-sections along the [$11\bar{2}$] showing the upper part of the hexagonal, 2-μm-wide and 8-μm-tall, initial Si pillar (gray) and the 3C-SiC crystal grown on top after 3 μm (red) and 6 μm (blue) of deposition. (**b**) Cross-section along the same direction for the simulated crystal: the black dashed lines are taken for each μm of deposition, the red (blue) profile corresponds to 3μm (6 μm) of deposition. (**c**) Simulated growth sequence in perspective view.

The morphologies observed in the two samples of Figure 2 were then considered as consecutive evolution stages (see Figure 3a) to be matched by a growth simulation based on the phase-field approach introduced in the Materials and Methods section. To this purpose, all possible facets, that is, {111}-Si and {111}-C terminated, {100} and {110}, are supposed to be local maximum for the $\tau (\hat{n})$ function so to result in local minima in the growth rate. Their relative values are tuned to best-fit the experimental profiles: $\tau_{100}$ = 0.55$\tau_{111C}$, $\tau_{111\mathrm{Si}}$ = 0.32$\tau_{111C}$, $\tau_{110}$ = 0.18$\tau_{111C}$ (see Figure 1b). An hexagonal prismatic pillar, bounded by {110} planes, is set as initial profile, sized as in the experiments and deposition is performed up to 6 μm.

Simulated profiles well reproduce the experimental behavior as made evident by the close correspondence of the calculated cross-sections in Figure 3b with the experimental ones of panel (a). The simulation sequence is also shown in 3D perspective view in Figure 3c to better appreciate the shape evolution. Notably, the SiC crystal expands laterally mostly above the top of the initial Si pillar, due to the larger amount of incoming material with respect to the sidewalls where a limited enlargement is yet present. The crystal develops quite vertically as the {111}-Si facet at the top advances faster then both {111}-C and {100} sides. At the same time, its perimeter changes from an hexagon delimited by <110> sides into a triangle with the three <110> sides in common with the {111}-C facet planes. This is direct consequence of the competition between {111}-C and {100} side facets. Indeed, at first both develop with similar extensions (2 μm SiC deposition). However, {111}-C facets have a much lower incorporation rate than {100} ones so that adatoms migrate on this latter enhancing their growth rate. As deposition proceeds, the difference in growth rate becomes more evident and {100} planes shrink in size, reducing to narrow stripes in between the large {111}-C facets (6 μm) and eventually disappear at later growth stages.

The compelling correspondence between simulations and experiments confirms the key role of anisotropic adatom kinetics in driving the morphological evolution, out-ruling the facet stability hierarchy expected on the basis of surface energy calculations at equilibrium. Moreover, at variance with the widely studied case of Ge crystals on Si pillars, which are reported to self-align vertically because of mutual flux shielding between neighbors [12,13], the SiC crystals are not apparently influenced by the proximity of other structures, even when close to each other (as in the case of Figure 2). Indeed, their morphological evolution seems fully determined by the surface diffusion and incorporation dynamics until they get in touch with each other.

Once demonstrated the reliability of simulations in reproducing known experiments, we can profitably exploit them for scouting new designs. A first aspect to be considered is the impact of the Si pillar shape set by the lithographic process. Previous simulation results were obtained for an hexagonal {110}-faceted prismatic pillar, as used in the experiments. This choice reflected the six-fold symmetry of the {111} Si substrate. However, the 3D SiC crystals have a three-fold symmetry with respect to the [111]-axis, resulting from the alternation of {111}-C and {100} planes on the sides. None of such facets has an edge in common with the {110} pillar sidewalls as they develop right in the middle. We then expect that the selected pillar shape does not influence the growth of the SiC facets letting them to develop in a natural way.

**Figure 4:**
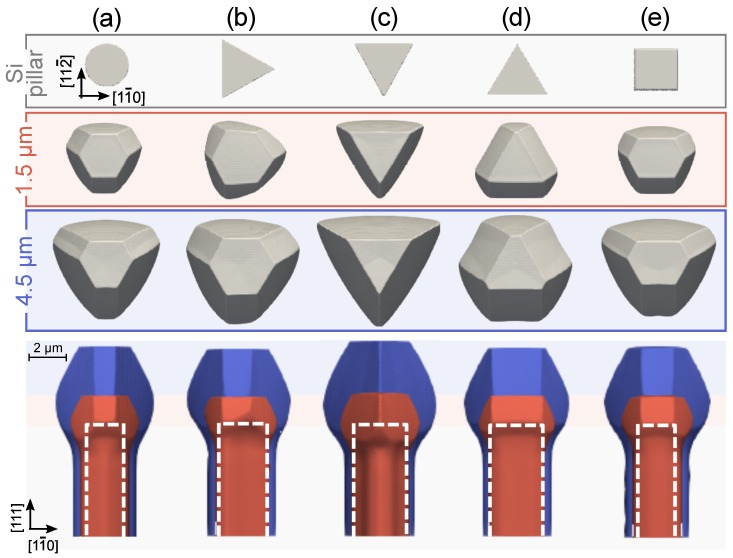
Simulations of crystal morphology evolution for different Si pillar shapes: (**a**) cylindrical, (**b**–**d**) prismatic with triangular base and (**e**) with squared base. Top views are reported after 1.5 and 4.5 μm of SiC deposition along with lateral views obtained by superposition of the corresponding stages: 1.5 μm in red and 4.5 μm in blue (the dashed white lines show the profile of the initial pillar in side view). In all cases, 7 μm tall Si pillars are considered with the same area of the top base, equal to 5 μm${}^{2}$.

Different alignments of the pillar facets can however favor or delay the nucleation of some of the SiC facets, distorting the crystal morphology as illustrated in the comparative chart of Figure 4 where different shapes and orientations have been considered for the Si pillar. In the case (a), a cylindrical Si pillar is considered. Similarly to the hexagonal prism of Figure 3, the crystal faceting arises spontaneously without resenting of the underlying structure.

This is no more the case when considering a prismatic shape with triangular base. In the case (b), sidewalls consist of {110} planes and have no edge in common with the SiC lateral facets. In the first stages of crystal growth (1.5 μm SiC) pairs of {111}-C and {100} facets with similar extension are formed on the edges of the Si triangular top, retaining the symmetry of the underlying Si pillar. However, as the growth proceeds, surface diffusion restores the unbalance in the facet growth rates discussed before, so that the slow {111}-C facets start dominating and the crystal morphology converges to the one obtained in (a), losing memory of the underlying Si pillar shape. The very same evolution is obtained for the specular triangular shape by symmetry. A bigger effect on the growth of the crystal can be instead obtained when rotating the triangular pillar by 90${}^{\circ }$ around the <111> vertical axis, so to have {112} sidewalls. Two different orientations are to be considered. In Figure 4c, the edges of the triangular top are common to the {111}-C facets of the above SiC crystal. Since such facets are also favored by the slow incorporation kinetics, they immediately develop on the Si top edges hindering the nucleation of the three {100} planes seen in the previous cases. The morphology of the growing SiC crystal then becomes that of a {111}-C pyramid, truncated by a {111}-Si top facet eventually shrinking in size for later stages. The opposite happens when taking the triangular base oriented as in the panel (d), that is, with edges in common with the {100} SiC facets. At the earliest stages of the growth (1.5 μm), the {100} facets develop all along the edges of the Si triangular base while small {111}-C planes nucleate at the corners. However, as deposition proceeds, the slow kinetics of {111}-C facets favors their enlargement by transferring adatoms toward the {100} ones. As the size of the {100} facets is initially defined by the pillar base, their area is enhanced with respect to the other cases in Figure 4 and comparable to that of {111}-C planes, even after deposition of several SiC microns (see 4.5 μm). Lateral views also reveal that case (c) returns a slightly taller crystal, due to the absence of {100} sides that in the other cases collect material from the neighboring {111} planes.

Notably, the templating effects identified for the (c) and (d) triangular cases are possible only because sidewalls are formed by only one of the two subsets of {112} facets perpendicular to the substrate. Indeed, if the Si pillar base were the hexagon formed by all six {112} sidewalls (not shown), the resulting SiC crystal morphology (not shown here) would not be much different from the one obtained with the most generic cylindrical shape since both {100} and {111}-C SiC facets would develop simultaneously on the edges of the Si top and hence compete in the same way.

A squared pillar shape is also considered in Figure 4e, formed by a pair of {110} and a pair of {112} sidewalls. Expectedly, at early growth stages (1.5 μm), the growing SiC crystal presents two elongated facets, a {111}-C and the opposite {100} in correspondence of the edges of the {112} sidewalls, while the other two {111}-C and two {100} facets are slightly shorter as the underlying Si pillar edges are not aligned with any of them requiring a certain material redistribution to make them apparent. This low-symmetry morphology closely resembles experimental results (not shown) obtained for squared pillars. For later stages, the spontaneous redistribution of material tends to restore the three-fold symmetry of panel (a), losing any templating effect of the underlying Si pillar.

All cases in Figure 4 were obtained for the same Si pillar-base area to enable a direct comparison. For the cases (a–c), as well as for hexagonal shapes, a variation in the Si pillar base area returns a self-similar shape evolution, eventually reproducing the here reported stages by a suitable scaling of deposition thicknesses. In the other cases (d–e), templating effects induce deviations in the initial SiC morphologies with respect to the “spontaneous” three-fold symmetry which are more pronounced and persistent when considering large bases.

**Figure 5:**
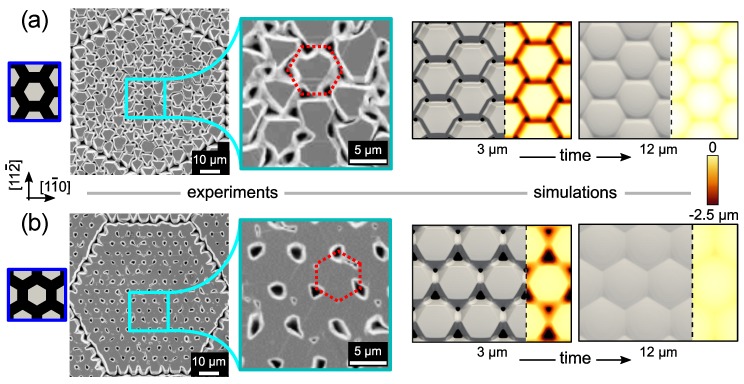
Top view of coalescence experiments for the deposition of 12 μm and simulations for two deposition stages. Two hexagonal patterns, sketched in the blue insets, are considered: (**a**) pillar rows along [11$\bar{2}$] and (**b**) pillar rows along [1$\bar{1}$0]. The surface height, refered to the (111) crystal top, is shown by the color maps. The Si pillar base is 5 μm and the gap between pillars is 2 μm.

So far we focused our attention on the growth of a single SiC crystal, as if it were isolated. However, Si pillars are arranged into dense arrays, spaced by just a few μm so that, by growing laterally, they can touch with each other and eventually coalesce into a continuous, suspended film (see Reference [8]). Simulations can be straightforwardly applied to model crystal coalescence since topological changes are naturally included in the evolution of the phase-field function. In Figure 5 we report two examples of crystal coalescence for hexagonal arrangements of the Si pillars, obtained by 90${}^{\circ }$ rotation of the same pattern around the [111]-axis, so to have pillar rows aligned in the [11$\bar{2}$] and [$\bar{1}$10] directions respectively (see insets). Experimental top views acquired after deposition of 12 μm SiC show in both cases a partially connected structure, consistently reproduced by the simulations (first stages reported in the figure). In pattern (a), the individual crystals touch with each other but they are still clearly distinguishable. {111}-C (larger) and {100} (smaller) side facets touch along the six {112} directions while leaving six empty holes in between. At later stages of deposition, simulations predict a progressive closure of the holes leaving trenches around the perimeter of each SiC crystal that could eventually smooth by further extension of the deposition process (or by prolonged high temperature annealing [21]). Actually, the quality of the films obtained in experiments does not improve much, probably because the trenches are formed by the contact of different facets. The presence of extensive step-bunchings on the facets at the contact region is also expected to have a role.

A flatter networked structure is instead observed when the growth is performed on the pattern (b). In this case, bridges are formed between facet edges. As well evident in the first stage of simulation and also distinguishable in the magnification of the SEM top view, two different arrays of holes are formed. Large ones appear in between the three {111}-C side facets of adjacent crystals while small ones are formed in between three {100} facets. By continuing deposition, as shown by the simulation, coalescence proceeds by the lateral expansion of the bridges. Then, the small holes get closed, followed by the largest ones, leaving small depressions on the top surface which are eventually filled. Surface roughness is lower than in the case with pattern (a) as it develops only in correspondence of the holes, returning a much better uniformity throughout the whole sample. Experiments where the growth was extended up to 18 μm SiC, as those in Reference [8], did show indeed almost planar, fully coalesced films.

The reconstruction of the entire evolution pathway from the pristine growth of individual crystals to the fully coalesced structure made possible by the simulations offers a more detailed view of the process. In particular, it is found that the onset of crystals coalescence is at around 2.5 μm SiC deposition in the case of pattern (a) while it is observed at just 2 μm for the pattern (b) due to the shorter distance between facet edges resulting by the crystal evolution in this latter arrangement. Also the subsequent filling of the holes is found to proceed faster for pattern (b) compared to the smoothing of the trenches formed in the case (a). Actually, the velocity of such smoothing processes is, in both cases, faster than what observed experimentally (see 12 μm in Figure 5). This is probably due to an overestimation of the growth rate in the concave regions, where a certain decrease in material supply is expected as their size shrinks.

## 4. Conclusions

It has been shown that the growth of SiC micro-crystals on Si pillars can be well explained by tackling adatom kinetics at the surface. The morphology evolution is indeed consistently reproduced by simulations accounting for facet-dependent incorporation times and looks quite different from an equilibrium crystal shape predictable by first-principle surface energy calculations.

With this respect, studying the 3D growth on Si pillars is a very convenient workbench for a more general characterization of the morphological traits of 3C-SiC crystals, hardly distinguishable in the more conventional planar growths or for irregular, poly-cristalline structures.

Simulations offered a great insight also on the effects on morphology produced by different pillar shapes and substrate orientations. In the first growth stages, crystal facets develop from the Si pillar base edges so that morphology tends to distort according to the underlying structure but, for later stages, the competition between facets restores the characteristic three-fold shape dominated by {111}-C planes. A stronger template effect is recognized only in the case of triangular bases with {112} sidewalls. Coalescence might be affected by such shape distortions only in the case of small inter-pillar distances or in the case of larger pillar sizes.

The crystallographic alignment of the pattern on the substrate is even more important to control the flatness of the suspended film, depending on the topology of the coalescence. The best results are achieved when pillar rows are along the <1$\bar{1}$0> direction, as the merging proceeds by the lateral expansion of bridges between the facet edges. On the contrary, the coalescence process results hampered when contact occurs between opposite {100} and {111}-C facets. The quality of the suspended film can then be improved by controlling the extent of the crystal facet at the point of coalescence by a careful definition of the pillar geometry and arrangement.
